# Crystal structure of KNaCuP_2_O_7_, a new member of the diphosphate family

**DOI:** 10.1107/S2056989018000130

**Published:** 2018-01-09

**Authors:** Ines Fitouri, Habib Boughzala

**Affiliations:** a Laboratoire de Materiaux et Cristallochimie, Faculté des Sciences de Tunis, Université de Tunis El Manar, 2092 Manar II Tunis, Tunisia

**Keywords:** crystal structure, diphosphate, eclipsed conformation, isotypism

## Abstract

Potassium sodium copper(II) diphosphate(V), KNaCuP_2_O_7_, was synthesized by solid-state reactions. It crystallizes in the *α*-Na_2_CuP_2_O_7_ structure type in space group *P*2_1_/*n*. In the crystal, CuO_5_ square-pyramids are linked to nearly eclipsed P_2_O_7_ groups by sharing corners to build up corrugated layers with composition [CuP_2_O_7_]^2−^ that extend parallel to (010). The K^+^ and Na^+^ cations reside in the inter­layer space and are connected to nine and seven O atoms, respectively. The structural model was validated by bond-valence-sum (BVS) and charge-distribution (CHARDI) analysis.

## Abstract 2

KNaCuP_2_O_7_ is a member of the diphosphate family and crystallizes isotypically with α-Na_2_CuP_2_O_7_. The structure exhibits nearly eclipsed diphosphate units, distorted CuO_5_ square-pyramids, and distorted NaO_7_ and KO_9_ polyhedra as the main building units.

## Chemical context   

In order to improve the ionic conductivity in copper diphosphates with general formula *MM*’CuP_2_O_7_ (*M, M*’ = monovalent cation), we attempted to partially replace the potassium cations in K_2_CuP_2_O_7_ by smaller sodium cations. In the K_2_CuP_2_O_7_ structure, the alkali cations are located in the inter­layer space between corrugated [CuP_2_O_7_]^2−^ anionic layers. Reducing the size of the cation can increase its mobility, and therefore might improve the material’s charge-transport behaviour.

Several attempts were made to prepare crystals of the hypothetical solid solution K_2–*x*_Na_*x*_CuP_2_O_7_, with *x* in the range 0 to 2. All of the attempts led to a compound with *x* = 1, *i.e.* KNaCuP_2_O_7_, the crystal structure of which is reported in this communication.

## Structural commentary   

**Figure 1:**
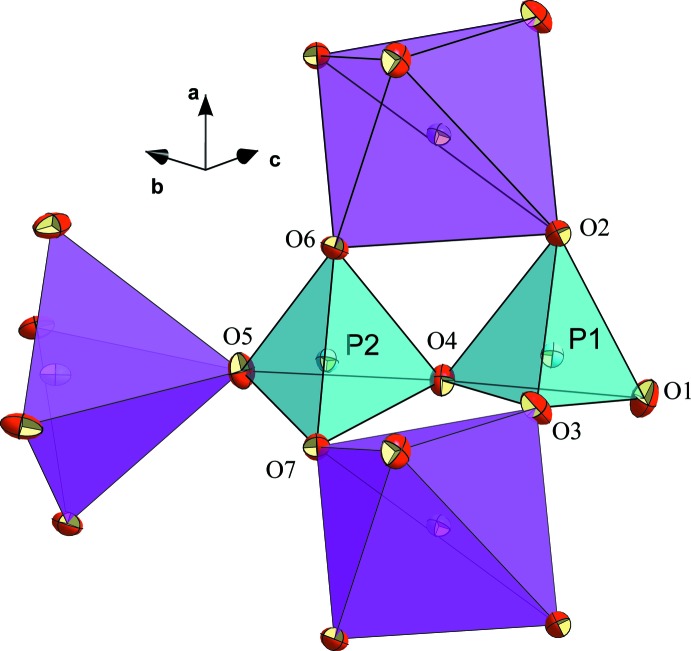
The diphosphate group directly connected to three CuO_5_ polyhedra in the structure of KNaCuP_2_O_7_. Displacement ellipsoids are drawn at the 50% probability level.

The title compound KNaCuP_2_O_7_ crystallizes isotypically with *α*-Na_2_CuP_2_O_7_ (Erragh *et al.*, 1995 ▸) and also shows resemblance with one form of K_2_CuP_2_O_7_ (ElMaadi *et al.*, 1995 ▸). It is built up by corrugated [CuP_2_O_7_]^2−^ layers with the alkali cations situated in the inter­layer space. The anionic layers consist of a nearly eclipsed diphosphate group [O2—P2—P1—O6 torsion angle = 15.90 (1)°] linked to CuO_5_ square-pyramids by sharing five of the terminal oxygen atoms (O2, O3, O5, O6, O7). The bridging atom O4 of the diphosphate unit is not involved in metal coordination, and atom O1 coordinates to the alkali cations in the inter­layer space (Fig. 1 ▸).

**Figure 2:**
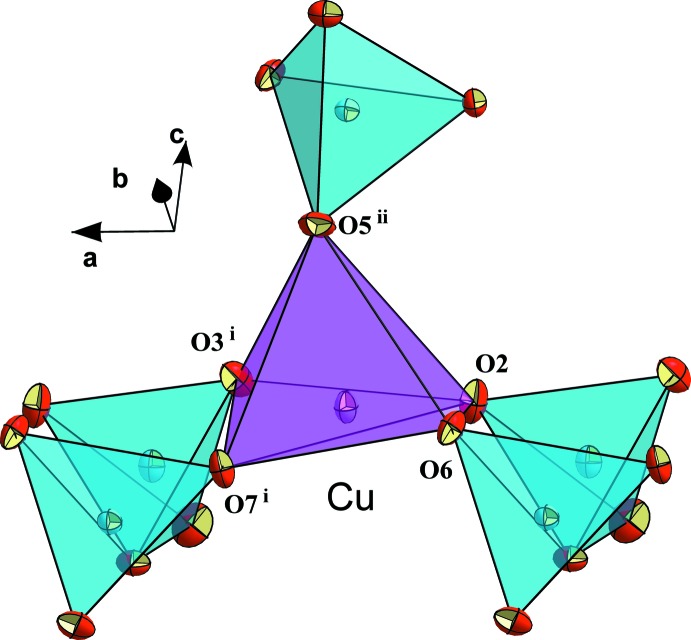
The CuO_5_ square-pyramid with neighbouring diphosphate groups in the structure of KNaCuP_2_O_7_. Displacement ellipsoids are drawn at the 50% probability level.

**Figure 3:**
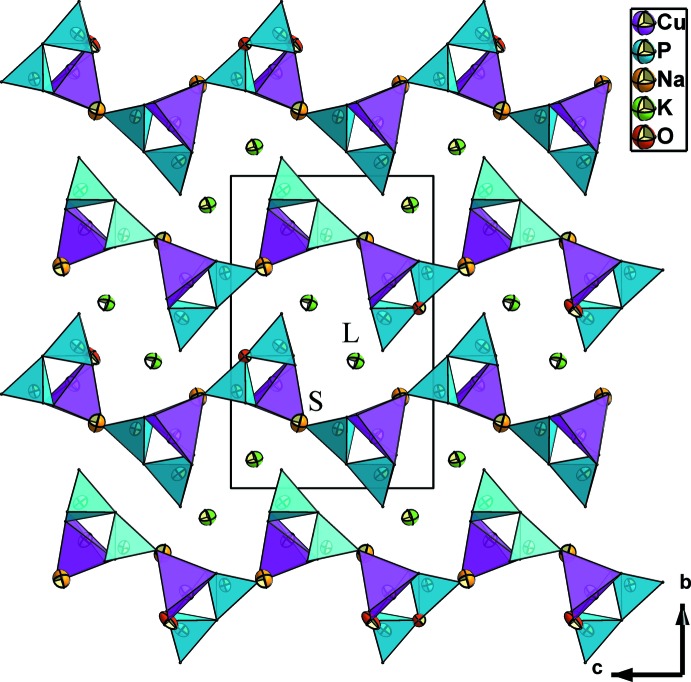
Projection of the KNaCuP_2_O_7_ structure along [100], showing the corrugated inter­layer space housing the cations K^+^ in the ‘*L*’ sites and Na^+^ in the ‘*S*’ sites. Displacement ellipsoids are drawn at the 99% probability level.

The P2—O4—P1 bridging angle [119.01 (11)°] of the diphosphate anion is close to those observed in other similar diarsenate and diphosphate structures, such as KCr_1/4_Al_3/4_As_2_O_7_ [118.50 (14)°; Bouhassine & Boughzala, 2017 ▸], CsCrAs_2_O_7_ [118.7 (2)°; Bouhassine & Boughzala, 2015 ▸], KAlAs_2_O_7_ [118.3 (2)°; Boughzala & Jouini, 1995 ▸], α-Na_2_CuP_2_O_7_ [118.65 (1)°; Erragh *et al.*, 1995 ▸] and K_2_CuP_2_O_7_ [120.41 (3)°; ElMaadi *et al.*, 1995 ▸]. As expected, the Cu—O bond length to the apical oxygen atom O5 is significantly longer than the Cu—O distances to the basal oxygen atoms of the square-pyramid (Table 1 ▸). The calculated bond-valence sum (Brown, 2002 ▸; Adams, 2003 ▸) of 1.94 valence units for the Cu site is in good agreement with the expected value of 2 for divalent copper. The geometry index of the CuO_5_ polyhedron *τ*_5_, as defined by Addison *et al.* (1984 ▸), has a value of 0.26, indicating a distorted square-pyramidal coordination environment (the value for an ideal square-pyramid is 0 while that of an ideal trigonal bipyramid is 1). Each CuO_5_ polyhedron shares its vertices with two P_2_O_7_^4−^ anions, one of which is chelating and the other belongs to two different P_2_O_7_ groups (Fig. 2 ▸). This linkage leads to layers extending parallel to (010) (Fig. 3 ▸).

**Figure 4:**
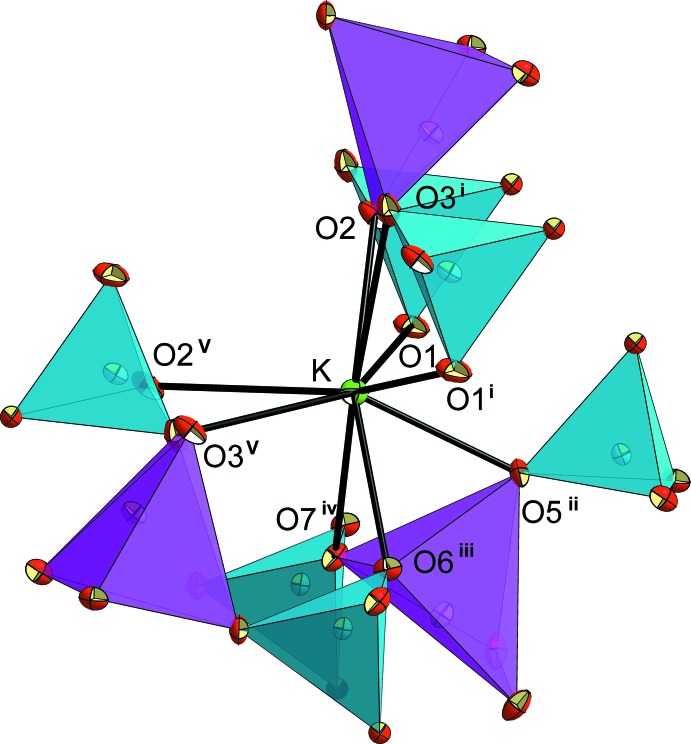
The nine-coordinated K^+^ cation in the large ‘*L*’ site within the inter­layer space in the structure of KNaCuP_2_O_7_. Displacement ellipsoids are drawn at 50% probability level. [Symmetry codes: (i) 1 + *x*, *y*, *z*; (ii) 1 − *x*, 1 − *y*, −*z*; (iii) 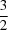 − *x*, −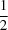 + *y*, 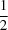 − *z*; (iv) 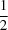 − *x*, −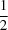 + *y*, 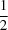 − *z*; (v) 1 − *x*, 1 − *y*, 1 − *z*.]

As in the crystal structures of K_2_CuP_2_O_7_ and *α*-Na_2_CuP_2_O_7_, the crystal structure of KNaCuP_2_O_7_ exhibits two independent sites hosting the K^+^ and Na^+^ cations. The first site is larger (‘*L*’) and is occupied by K^+^ cations. It is limited by two neighbouring anionic layers (Fig. 3 ▸). The resulting KO_9_ coord­ination polyhedron is considerably distorted (Fig. 4 ▸, Table 1 ▸). Its bond-valence sum is 0.98 valence units (Table 2 ▸). The second site is smaller (‘*S*’) and is occupied by Na^+^ cations. It is surrounded by three CuO_5_ and five PO_4_ polyhedra, delimiting an ellipsoidal cavity as shown in Figs. 3 ▸ and 5 ▸. The irregular coordination sphere of Na^+^ is made up of seven oxygen atoms and shows an effective coordination number (ECoN; Nespolo *et al.*, 2001 ▸) of 5.2 (for other ECoN values, see Table 2 ▸). The Na—O bonds lengths can be divided in groups of four short [2.249 (2)–2.4442 (19) Å] and three long [2.772 (2)–2.878 (2) Å] bonds (Table 1 ▸). Its bond-valence sum is 0.98 valence units (Table 2 ▸).

## Database survey   

Table 3 ▸ summarizes lattice parameters and the symmetry of related *MM*’CuP_2_O_7_ (*M, M*’ = monovalent cation) compounds compiled in the ICSD (ICSD, 2017 ▸). It is apparent that the size of the cation(s) defines the structure type.

## Synthesis and crystallization   

Crystals of KNaCuP_2_O_7_ were obtained from a mixture of KH_2_PO_4_, NaH_2_PO_4_ and CuO in the molar ratio K:Na:Cu:P = 1:1:2:2. The stoichiometric mixture was finely ground and heated in a porcelain crucible at 623 K for 12 h to eliminate volatile products. The temperature was then increased to 873 K and held for 15 d until fusion was reached. The sample was slowly cooled (5 K d^−1^) to 500 K and finally allowed to cool radiatively to room temperature. The product was washed with water and rinsed with an aqueous solution of HCl (low concentration). Only one type of regular light-blue prismatic crystals was observed. The obtained crystals were ground and checked by powder X-ray diffraction. Rietveld analysis with the program *TOPAS 4.2* (Coelho, 2009 ▸) revealed a single-phase product of KNaCuP_2_O_7_.

## Refinement   

Crystal data, data collection and structure refinement details are summarized in Table 4 ▸. The occupancy of the Na^+^ and K^+^ sites was checked by independent refinement of the site occupation factors. In each case, full occupancy was observed without the contribution of the other cation. The maximum and minimum electron densities remaining in the difference-Fourier map are located 0.67 Å from O7 and 0.48 Å from Cu, respectively.

## Supplementary Material

Crystal structure: contains datablock(s) I. DOI: 10.1107/S2056989018000130/wm5429sup1.cif

Structure factors: contains datablock(s) I. DOI: 10.1107/S2056989018000130/wm5429Isup2.hkl

CCDC reference: 1814470

Additional supporting information:  crystallographic information; 3D view; checkCIF report

## Figures and Tables

**Figure 5:**
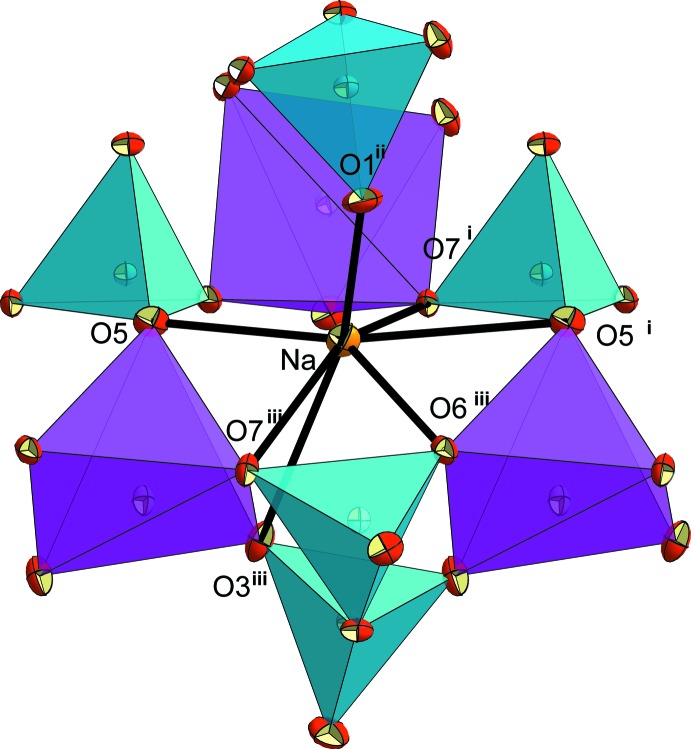
The surrounding of the seven-coordinated Na^+^ cation in the ‘*S*’ site in the structure of KNaCuP_2_O_7_. Displacement ellipsoids are drawn at 50% probability level. [Symmetry codes: (i) 1 + *x*, *y*, *z*; (ii) 1 − *x*, 1 − *y*, −*z*; (iii) 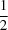 + *x*, 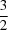 − *y*, −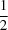 + *z*].

**Table 1:** Selected bond lengths (Å)

Cu—O2	1.9328 (18)	Na—O5	2.398 (2)
Cu—O3^i^	1.9427 (18)	Na—O7^i^	2.4442 (19)
Cu—O6	1.9743 (17)	Na—O3^iv^	2.772 (2)
Cu—O7^i^	1.9872 (17)	Na—O5^i^	2.815 (2)
Cu—O5^ii^	2.3225 (19)	Na—O7^iv^	2.878 (2)
P1—O1	1.482 (2)	K—O2	2.721 (2)
P1—O2	1.5246 (19)	K—O5^iii^	2.7245 (18)
P1—O3	1.5313 (19)	K—O1^i^	2.764 (2)
P1—O4	1.6272 (17)	K—O6^v^	2.7969 (19)
P2—O5	1.4958 (17)	K—O7^vi^	2.8450 (19)
P2—O6	1.5252 (17)	K—O3^vii^	2.8630 (18)
P2—O7	1.5277 (16)	K—O1	3.036 (2)
P2—O4	1.6148 (18)	K—O2^vii^	3.1973 (19)
Na—O1^iii^	2.249 (2)	K—O3^i^	3.257 (2)
Na—O6^iv^	2.397 (2)		

Symmetry codes: (i) 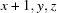; (ii) 
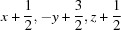
; (iii) 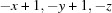; (iv) 
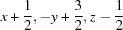
; (v) 
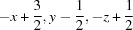
; (vi) 
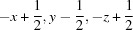
; (vii) 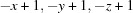.

**Table 2:** CHARDI and BVS analysis of cation polyhedra in KNaCuP_2_O_7_

Cation	*qi*()·sof(*i*)	*Q*(*i*)	*V*(*i*)·sof(*i*)	CN(*i*)	ECoN(*i*)	*d*_ar_(*i*)	*d*_med_(*i*)
Cu	2.000	1.94	1.995	5	4.35	2.031	2.031
P1	5.000	5.07	4.921	4	3.84	1.540	1.541
P2	5.000	5.03	4.938	4	3.88	1.540	1.540
K	1.000	0.98	1.103	9	5.20	2.622	2.564
Na	1.000	0.98	1.106	7	7.80	2.912	2.912

Notes: *qi* = formal oxydation number; sof(*i*) = site occupation factor; *d*_ar_(*i*) = average distance; *d*_med_(*i*) = weighted average distance; CN(*i*) = coordination number; ECoN(*i*)= effective coordination number; σ_cat_ = dispersion factor on cationic charges measuring the deviation of the computed charges; σ_cat_=[Σ_*i*_(*q_i_* − *Q_i_*)^2^/*N* − 1]^1/2^ = 0.019.

**Table 3:** Structural data (Å, °) for the *M*,*M*′_2_CuP_2_O_7_ family of compounds

Compound	Space group	*a*	*b*	*c*	*β*	*Z*	Reference
Li_2_CuP_2_O_7_	*I*2/*a*	14.068 (2)	4.8600 (8)	8.604 (1)	98.97 (1)	4	Spirlet *et al.* (1993 ▸)
Li_2_CuP_2_O_7_	*C*2/*c*	15.3360 (14)	4.8733 (13)	8.6259 (16)	114.795 (10)	4	Gopalakrishna *et al.* (2008 ▸)
α-Na_2_CuP_2_O_7_	*P*2_1_/*n*	8.823 (3)	13.494 (3)	5.108 (2)	92.77 (3)	4	Erragh *et al.* (1995 ▸)
β-Na_2_CuP_2_O_7_	*C*2/*c*	14.715 (1)	5.704 (2)	8.066 (1)	115.14 (1)	4	Etheredge *et al.* (1995 ▸)
K_2_CuP_2_O_7_	*Pbnm*	9.509 (4)	14.389 (6)	5.276 (2)		4	ElMaadi *et al.* (1995 ▸)
K_2_CuP_2_O_7_	*P* 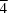 2_1_ *m*	8.056 (2)		5.460 (11)		2	Keates *et al.* (2014 ▸)
α-Rb_2_CuP_2_O_7_	*Pmcn*	5.183 (1)	10.096 (1)	15.146 (2)		4	Chernyatieva *et al.* (2008 ▸)
β-Rb_2_CuP_2_O_7_	*Cc*	7,002 (1)	12.751 (3)	9.773 (2)	110.93 (3)	4	Shvanskaya *et al.* (2012 ▸)
Cs_2_CuP_2_O_7_	*Cc*	7.460 (6)	12.973 (10)	9.980 (8)	111.95	4	Mannasova *et al.* (2016 ▸)
NaCsCuP_2_O_7_	*Pmn*2_1_	5.147 (2)	15.126 (3)	9.717 (5)		4	Chernyatieva *et al.* (2009 ▸)
Na_1.12_Ag_0.88_CuP_2_O_7_	*C*2/*c*	15.088 (2)	5.641 (1)	8.171 (1)	116.11 (1)	4	Bennazha *et al.* (2002 ▸)

**Table 4:** Experimental details

Crystal data	
Chemical formula	KNaCuP_2_O_7_
*M*_r_	299.57
Crystal system, space group	Monoclinic, *P*2_1_/*n*
Temperature (K)	298
*a*, *b*, *c* (Å)	5.176 (3), 13.972 (5), 9.067 (3)
β (°)	91.34 (2)
*V* (Å^3^)	655.6 (5)
*Z*	4
Radiation type	Mo *K*α
μ (mm^−1^)	4.51
Crystal size (mm)	0.18 × 0.13 × 0.09

Data collection	
Diffractometer	Enraf–Nonius CAD-4
Absorption correction	ψ scan (North *et al.*, 1968 ▸)
*T*_min_, *T*_max_	0.868, 0.997
No. of measured, independent and observed [*I* > 2σ(*I*)] reflections	3300, 1425, 1291
*R*_int_	0.036
(sin θ/λ)_max_ (Å^−1^)	0.638

Refinement	
*R*[*F* ^2^ > 2σ(*F* ^2^)], *wR*(*F* ^2^), *S*	0.020, 0.059, 1.01
No. of reflections	1425
No. of parameters	110
Δρ_max_, Δρ_min_ (e Å^−3^)	0.48, −0.42

Computer programs: *CAD-4 EXPRESS* (Enraf–Nonius, 1994 ▸), *XCAD4* (Harms & Wocadlo, 1995 ▸), *SHELXS97* (Sheldrick, 2008 ▸), *SHELXL2014/7* (Sheldrick, 2015 ▸), *DIAMOND* (Brandenburg, 2008 ▸) and *publCIF* (Westrip, 2010 ▸).

## supplementary crystallographic information

### Crystal data

**Table:**

KNaCuP_2_O_7_	*F*(000) = 580
*M**_r_* = 299.57	*D*_x_ = 3.035 Mg m^−^^3^
Monoclinic, *P*2_1_/*n*	Mo *K*α radiation, λ = 0.71073 Å
*a* = 5.176 (3) Å	Cell parameters from 25 reflections
*b* = 13.972 (5) Å	θ = 10–15°
*c* = 9.067 (3) Å	µ = 4.51 mm^−^^1^
β = 91.34 (2)°	*T* = 298 K
*V* = 655.6 (5) Å^3^	Prism, blue
*Z* = 4	0.18 × 0.13 × 0.09 mm

### Data collection

**Table:**

Enraf–Nonius CAD-4 diffractometer	*R*_int_ = 0.036
Radiation source: fine-focus sealed tube	θ_max_ = 27.0°, θ_min_ = 2.7°
ω/2θ scans	*h* = −6→6
Absorption correction: ψ scan (North *et al.*, 1968)	*k* = −2→17
*T*_min_ = 0.868, *T*_max_ = 0.997	*l* = −11→11
3300 measured reflections	2 standard reflections every 120 min
1425 independent reflections	intensity decay: 1.1%
1291 reflections with *I* > 2σ(*I*)	

### Refinement

**Table:**

Refinement on *F*^2^	Primary atom site location: structure-invariant direct methods
Least-squares matrix: full	Secondary atom site location: difference Fourier map
*R*[*F*^2^ > 2σ(*F*^2^)] = 0.020	*w* = 1/[σ^2^(*F*_o_^2^) + (0.0261*P*)^2^ + 0.7316*P*] where *P* = (*F*_o_^2^ + 2*F*_c_^2^)/3
*wR*(*F*^2^) = 0.059	(Δ/σ)_max_ = 0.001
*S* = 1.01	Δρ_max_ = 0.48 e Å^−^^3^
1425 reflections	Δρ_min_ = −0.42 e Å^−^^3^
110 parameters	Extinction correction: SHELXL-2014/7 (Sheldrick, 2015), Fc^*^=kFc[1+0.001xFc^2^λ^3^/sin(2θ)]^-1/4^
0 restraints	Extinction coefficient: 0.0120 (9)

### Special details

**Table:**

Geometry. All esds (except the esd in the dihedral angle between two l.s. planes) are estimated using the full covariance matrix. The cell esds are taken into account individually in the estimation of esds in distances, angles and torsion angles; correlations between esds in cell parameters are only used when they are defined by crystal symmetry. An approximate (isotropic) treatment of cell esds is used for estimating esds involving l.s. planes.
Refinement. Refinement of F^2^ against ALL reflections. The weighted R-factor wR and goodness of fit S are based on F^2^, conventional R-factors R are based on F, with F set to zero for negative F^2^. The threshold expression of F^2^ > 2sigma(F^2^) is used only for calculating R-factors(gt) etc. and is not relevant to the choice of reflections for refinement. R-factors based on F^2^ are statistically about twice as large as those based on F, and R- factors based on ALL data will be even larger.

### Fractional atomic coordinates and isotropic or equivalent isotropic displacement parameters (Å^2^)

**Table:**

	*x*	*y*	*z*	*U*_iso_*/*U*_eq_	
Cu	0.76039 (5)	0.66590 (2)	0.21888 (3)	0.01093 (12)	
P1	0.26527 (12)	0.54589 (5)	0.24314 (7)	0.01115 (15)	
P2	0.25245 (11)	0.68972 (4)	0.02910 (6)	0.00898 (15)	
Na	0.7521 (2)	0.70912 (8)	−0.16272 (11)	0.0192 (2)	
K	0.75717 (11)	0.40697 (4)	0.38439 (6)	0.01850 (15)	
O1	0.2223 (4)	0.44115 (15)	0.2502 (2)	0.0223 (4)	
O2	0.5278 (3)	0.57557 (14)	0.3077 (2)	0.0176 (4)	
O3	0.0576 (3)	0.60643 (15)	0.31633 (19)	0.0179 (4)	
O4	0.2588 (3)	0.57729 (12)	0.07029 (18)	0.0126 (4)	
O5	0.2938 (3)	0.69884 (14)	−0.13300 (18)	0.0160 (4)	
O6	0.4685 (3)	0.73465 (13)	0.12308 (19)	0.0138 (4)	
O7	−0.0125 (3)	0.72712 (13)	0.07246 (19)	0.0139 (4)	

### Atomic displacement parameters (Å^2^)

**Table:**

	*U*^11^	*U*^22^	*U*^33^	*U*^12^	*U*^13^	*U*^23^
Cu	0.00808 (16)	0.01226 (18)	0.01244 (17)	0.00043 (11)	0.00010 (11)	0.00296 (11)
P1	0.0103 (3)	0.0102 (3)	0.0130 (3)	0.0000 (2)	0.0007 (2)	0.0029 (2)
P2	0.0088 (3)	0.0099 (3)	0.0082 (3)	0.0002 (2)	0.0008 (2)	0.0009 (2)
Na	0.0190 (5)	0.0213 (6)	0.0172 (5)	−0.0021 (4)	−0.0001 (4)	0.0020 (5)
K	0.0220 (3)	0.0153 (3)	0.0182 (3)	0.0022 (2)	−0.0008 (2)	0.0000 (2)
O1	0.0272 (10)	0.0115 (10)	0.0280 (10)	−0.0046 (8)	0.0001 (8)	0.0049 (8)
O2	0.0123 (8)	0.0198 (10)	0.0206 (9)	−0.0026 (8)	−0.0022 (7)	0.0093 (8)
O3	0.0151 (9)	0.0261 (10)	0.0126 (8)	0.0075 (8)	0.0008 (7)	0.0036 (8)
O4	0.0178 (8)	0.0093 (8)	0.0107 (8)	−0.0006 (7)	0.0012 (6)	−0.0003 (7)
O5	0.0213 (9)	0.0179 (9)	0.0088 (8)	0.0015 (8)	0.0025 (7)	0.0013 (7)
O6	0.0115 (8)	0.0115 (8)	0.0181 (8)	−0.0002 (7)	−0.0041 (6)	0.0011 (7)
O7	0.0091 (8)	0.0154 (9)	0.0173 (8)	0.0016 (7)	0.0030 (6)	0.0044 (8)

### Geometric parameters (Å, º)

**Table:**

Cu—O2	1.9328 (18)	Na—P2^i^	3.0977 (12)
Cu—O3^i^	1.9427 (18)	Na—P2^viii^	3.1316 (12)
Cu—O6	1.9743 (17)	Na—Cu^ix^	3.2481 (11)
Cu—O7^i^	1.9872 (17)	Na—Cu^viii^	3.3550 (11)
Cu—O5^ii^	2.3225 (19)	K—O2	2.721 (2)
Cu—Na^ii^	3.2481 (11)	K—O5^vii^	2.7245 (18)
Cu—Na^iii^	3.3550 (11)	K—O1^i^	2.764 (2)
Cu—K^iv^	3.4966 (7)	K—O6^x^	2.7969 (19)
Cu—Na	3.5117 (10)	K—O7^xi^	2.8450 (19)
Cu—K	3.9169 (7)	K—O3^v^	2.8630 (18)
P1—O1	1.482 (2)	K—O1	3.036 (2)
P1—O2	1.5246 (19)	K—O2^v^	3.1973 (19)
P1—O3	1.5313 (19)	K—O3^i^	3.257 (2)
P1—O4	1.6272 (17)	K—P1^v^	3.4455 (9)
P1—K	3.4260 (9)	K—Cu^x^	3.4965 (7)
P1—K^v^	3.4455 (9)	O1—Na^vii^	2.249 (2)
P1—K^vi^	3.5329 (9)	O1—K^vi^	2.764 (2)
P2—O5	1.4958 (17)	O2—K^v^	3.1973 (19)
P2—O6	1.5252 (17)	O3—Cu^vi^	1.9427 (18)
P2—O7	1.5277 (16)	O3—Na^iii^	2.772 (2)
P2—O4	1.6148 (18)	O3—K^v^	2.8630 (18)
P2—Na^vi^	3.0977 (12)	O3—K^vi^	3.257 (2)
P2—Na^iii^	3.1316 (12)	O5—Cu^ix^	2.3225 (19)
P2—Na	3.1617 (12)	O5—K^vii^	2.7245 (18)
Na—O1^vii^	2.249 (2)	O5—Na^vi^	2.815 (2)
Na—O6^viii^	2.397 (2)	O6—Na^iii^	2.397 (2)
Na—O5	2.398 (2)	O6—K^iv^	2.7968 (19)
Na—O7^i^	2.4442 (19)	O7—Cu^vi^	1.9872 (17)
Na—O3^viii^	2.772 (2)	O7—Na^vi^	2.4441 (19)
Na—O5^i^	2.815 (2)	O7—K^xii^	2.8451 (19)
Na—O7^viii^	2.878 (2)	O7—Na^iii^	2.878 (2)
			
O2—Cu—O3^i^	91.46 (8)	O5—Na—Cu^ix^	45.56 (5)
O2—Cu—O6	91.34 (7)	O7^i^—Na—Cu^ix^	127.14 (6)
O3^i^—Cu—O6	176.22 (8)	O3^viii^—Na—Cu^ix^	36.58 (4)
O2—Cu—O7^i^	160.62 (8)	O5^i^—Na—Cu^ix^	146.13 (6)
O3^i^—Cu—O7^i^	90.77 (7)	O7^viii^—Na—Cu^ix^	37.24 (4)
O6—Cu—O7^i^	87.43 (7)	P2^i^—Na—Cu^ix^	150.57 (4)
O2—Cu—O5^ii^	109.22 (7)	P2^viii^—Na—Cu^ix^	58.62 (2)
O3^i^—Cu—O5^ii^	92.15 (8)	P2—Na—Cu^ix^	65.37 (2)
O6—Cu—O5^ii^	84.52 (7)	O1^vii^—Na—Cu^viii^	108.65 (6)
O7^i^—Cu—O5^ii^	89.93 (7)	O6^viii^—Na—Cu^viii^	35.44 (4)
O2—Cu—Na^ii^	134.76 (6)	O5—Na—Cu^viii^	148.47 (6)
O3^i^—Cu—Na^ii^	58.24 (7)	O7^i^—Na—Cu^viii^	81.19 (5)
O6—Cu—Na^ii^	118.00 (6)	O3^viii^—Na—Cu^viii^	77.32 (5)
O7^i^—Cu—Na^ii^	61.20 (5)	O5^i^—Na—Cu^viii^	43.13 (4)
O5^ii^—Cu—Na^ii^	47.48 (5)	O7^viii^—Na—Cu^viii^	86.18 (4)
O2—Cu—Na^iii^	72.89 (6)	P2^i^—Na—Cu^viii^	64.76 (2)
O3^i^—Cu—Na^iii^	134.08 (6)	P2^viii^—Na—Cu^viii^	57.50 (2)
O6—Cu—Na^iii^	44.76 (5)	P2—Na—Cu^viii^	151.44 (4)
O7^i^—Cu—Na^iii^	118.06 (6)	Cu^ix^—Na—Cu^viii^	103.22 (3)
O5^ii^—Cu—Na^iii^	55.94 (5)	O2—K—O5^vii^	102.87 (6)
Na^ii^—Cu—Na^iii^	103.22 (3)	O2—K—O1^i^	96.73 (6)
O2—Cu—K^iv^	136.72 (6)	O5^vii^—K—O1^i^	78.10 (6)
O3^i^—Cu—K^iv^	123.31 (6)	O2—K—O6^x^	163.30 (6)
O6—Cu—K^iv^	53.04 (5)	O5^vii^—K—O6^x^	63.38 (5)
O7^i^—Cu—K^iv^	54.45 (5)	O1^i^—K—O6^x^	71.95 (6)
O5^ii^—Cu—K^iv^	51.11 (5)	O2—K—O7^xi^	127.36 (6)
Na^ii^—Cu—K^iv^	65.54 (2)	O5^vii^—K—O7^xi^	66.49 (5)
Na^iii^—Cu—K^iv^	64.45 (2)	O1^i^—K—O7^xi^	127.46 (6)
O2—Cu—Na	121.97 (6)	O6^x^—K—O7^xi^	58.05 (5)
O3^i^—Cu—Na	120.87 (5)	O2—K—O3^v^	115.68 (6)
O6—Cu—Na	59.42 (5)	O5^vii^—K—O3^v^	141.38 (6)
O7^i^—Cu—Na	42.40 (5)	O1^i^—K—O3^v^	98.78 (6)
O5^ii^—Cu—Na	115.40 (5)	O6^x^—K—O3^v^	78.91 (6)
Na^ii^—Cu—Na	102.98 (2)	O7^xi^—K—O3^v^	87.26 (6)
Na^iii^—Cu—Na	103.54 (2)	O2—K—O1	51.17 (5)
K^iv^—Cu—Na	64.59 (2)	O5^vii^—K—O1	71.36 (6)
O2—Cu—K	39.55 (6)	O1^i^—K—O1	126.29 (7)
O3^i^—Cu—K	56.02 (6)	O6^x^—K—O1	125.76 (6)
O6—Cu—K	127.43 (5)	O7^xi^—K—O1	77.84 (5)
O7^i^—Cu—K	131.41 (5)	O3^v^—K—O1	132.24 (6)
O5^ii^—Cu—K	122.07 (5)	O2—K—O2^v^	87.12 (5)
Na^ii^—Cu—K	112.35 (2)	O5^vii^—K—O2^v^	137.25 (6)
Na^iii^—Cu—K	110.35 (2)	O1^i^—K—O2^v^	142.70 (6)
K^iv^—Cu—K	172.759 (11)	O6^x^—K—O2^v^	109.37 (5)
Na—Cu—K	122.43 (2)	O7^xi^—K—O2^v^	74.42 (5)
O1—P1—O2	112.67 (12)	O3^v^—K—O2^v^	47.81 (5)
O1—P1—O3	114.78 (12)	O1—K—O2^v^	84.43 (5)
O2—P1—O3	108.17 (11)	O2—K—O3^i^	54.42 (5)
O1—P1—O4	107.95 (11)	O5^vii^—K—O3^i^	110.06 (5)
O2—P1—O4	107.13 (10)	O1^i^—K—O3^i^	49.03 (5)
O3—P1—O4	105.64 (10)	O6^x^—K—O3^i^	119.15 (5)
O1—P1—K	62.31 (8)	O7^xi^—K—O3^i^	176.12 (5)
O2—P1—K	50.40 (8)	O3^v^—K—O3^i^	94.86 (5)
O3—P1—K	132.38 (7)	O1—K—O3^i^	102.97 (5)
O4—P1—K	120.75 (7)	O2^v^—K—O3^i^	109.40 (5)
O1—P1—K^v^	97.92 (9)	O2—K—P1	25.58 (4)
O2—P1—K^v^	67.78 (7)	O5^vii^—K—P1	86.44 (4)
O3—P1—K^v^	55.22 (7)	O1^i^—K—P1	112.63 (5)
O4—P1—K^v^	153.15 (7)	O6^x^—K—P1	148.42 (4)
K—P1—K^v^	77.51 (2)	O7^xi^—K—P1	102.86 (4)
O1—P1—K^vi^	47.78 (8)	O3^v^—K—P1	128.38 (4)
O2—P1—K^vi^	132.43 (7)	O1—K—P1	25.61 (4)
O3—P1—K^vi^	67.06 (8)	O2^v^—K—P1	85.94 (4)
O4—P1—K^vi^	119.91 (7)	O3^i^—K—P1	78.37 (3)
K—P1—K^vi^	96.10 (2)	O2—K—P1^v^	93.47 (4)
K^v^—P1—K^vi^	72.974 (19)	O5^vii^—K—P1^v^	156.78 (4)
O5—P2—O6	113.20 (10)	O1^i^—K—P1^v^	116.62 (5)
O5—P2—O7	111.96 (10)	O6^x^—K—P1^v^	102.54 (4)
O6—P2—O7	111.49 (10)	O7^xi^—K—P1^v^	90.50 (4)
O5—P2—O4	107.91 (10)	O3^v^—K—P1^v^	26.06 (4)
O6—P2—O4	105.10 (10)	O1—K—P1^v^	108.15 (4)
O7—P2—O4	106.66 (10)	O2^v^—K—P1^v^	26.19 (3)
O5—P2—Na^vi^	65.03 (7)	O3^i^—K—P1^v^	92.83 (4)
O6—P2—Na^vi^	150.48 (8)	P1—K—P1^v^	102.49 (2)
O7—P2—Na^vi^	51.00 (7)	O2—K—Cu^x^	139.21 (4)
O4—P2—Na^vi^	103.13 (7)	O5^vii^—K—Cu^x^	41.57 (4)
O5—P2—Na^iii^	147.30 (9)	O1^i^—K—Cu^x^	93.78 (5)
O6—P2—Na^iii^	48.05 (7)	O6^x^—K—Cu^x^	34.34 (3)
O7—P2—Na^iii^	66.21 (7)	O7^xi^—K—Cu^x^	34.63 (3)
O4—P2—Na^iii^	103.48 (7)	O3^v^—K—Cu^x^	101.36 (5)
Na^vi^—P2—Na^iii^	116.33 (3)	O1—K—Cu^x^	91.51 (4)
O5—P2—Na	46.71 (7)	O2^v^—K—Cu^x^	107.32 (4)
O6—P2—Na	70.95 (7)	O3^i^—K—Cu^x^	141.53 (4)
O7—P2—Na	149.66 (7)	P1—K—Cu^x^	115.54 (2)
O4—P2—Na	101.43 (7)	P1^v^—K—Cu^x^	116.45 (2)
Na^vi^—P2—Na	111.56 (3)	P1—O1—Na^vii^	153.68 (14)
Na^iii^—P2—Na	118.02 (3)	P1—O1—K^vi^	108.83 (11)
O1^vii^—Na—O6^viii^	89.29 (8)	Na^vii^—O1—K^vi^	93.05 (7)
O1^vii^—Na—O5	92.89 (8)	P1—O1—K	92.08 (9)
O6^viii^—Na—O5	126.37 (7)	Na^vii^—O1—K	86.20 (7)
O1^vii^—Na—O7^i^	111.80 (8)	K^vi^—O1—K	126.29 (7)
O6^viii^—Na—O7^i^	116.12 (7)	P1—O2—Cu	125.16 (11)
O5—Na—O7^i^	112.48 (7)	P1—O2—K	104.02 (10)
O1^vii^—Na—O3^viii^	150.23 (8)	Cu—O2—K	113.55 (8)
O6^viii^—Na—O3^viii^	79.37 (6)	P1—O2—K^v^	86.03 (8)
O5—Na—O3^viii^	72.83 (6)	Cu—O2—K^v^	128.21 (9)
O7^i^—Na—O3^viii^	97.87 (7)	K—O2—K^v^	92.88 (5)
O1^vii^—Na—O5^i^	85.37 (7)	P1—O3—Cu^vi^	126.56 (11)
O6^viii^—Na—O5^i^	67.10 (6)	P1—O3—Na^iii^	106.61 (10)
O5—Na—O5^i^	166.46 (9)	Cu^vi^—O3—Na^iii^	85.17 (8)
O7^i^—Na—O5^i^	56.39 (5)	P1—O3—K^v^	98.72 (8)
O3^viii^—Na—O5^i^	114.42 (7)	Cu^vi^—O3—K^v^	134.68 (8)
O1^vii^—Na—O7^viii^	91.46 (7)	Na^iii^—O3—K^v^	83.27 (6)
O6^viii^—Na—O7^viii^	56.27 (5)	P1—O3—K^vi^	87.28 (9)
O5—Na—O7^viii^	70.10 (6)	Cu^vi^—O3—K^vi^	94.34 (7)
O7^i^—Na—O7^viii^	156.06 (5)	Na^iii^—O3—K^vi^	163.09 (7)
O3^viii^—Na—O7^viii^	59.34 (5)	K^v^—O3—K^vi^	85.14 (5)
O5^i^—Na—O7^viii^	123.32 (6)	P2—O4—P1	119.01 (11)
O1^vii^—Na—P2^i^	93.49 (6)	P2—O5—Cu^ix^	128.83 (11)
O6^viii^—Na—P2^i^	94.77 (5)	P2—O5—Na	106.28 (10)
O5—Na—P2^i^	138.41 (6)	Cu^ix^—O5—Na	86.95 (7)
O7^i^—Na—P2^i^	29.06 (4)	P2—O5—K^vii^	139.70 (11)
O3^viii^—Na—P2^i^	114.67 (5)	Cu^ix^—O5—K^vii^	87.32 (5)
O5^i^—Na—P2^i^	28.80 (4)	Na—O5—K^vii^	90.88 (6)
O7^viii^—Na—P2^i^	150.56 (5)	P2—O5—Na^vi^	86.16 (8)
O1^vii^—Na—P2^viii^	96.04 (6)	Cu^ix^—O5—Na^vi^	80.94 (6)
O6^viii^—Na—P2^viii^	28.24 (4)	Na—O5—Na^vi^	166.46 (9)
O5—Na—P2^viii^	98.55 (5)	K^vii^—O5—Na^vi^	82.58 (6)
O7^i^—Na—P2^viii^	136.17 (6)	P2—O6—Cu	126.10 (11)
O3^viii^—Na—P2^viii^	61.77 (4)	P2—O6—Na^iii^	103.71 (9)
O5^i^—Na—P2^viii^	94.99 (5)	Cu—O6—Na^iii^	99.80 (7)
O7^viii^—Na—P2^viii^	29.06 (3)	P2—O6—K^iv^	134.97 (10)
P2^i^—Na—P2^viii^	121.50 (4)	Cu—O6—K^iv^	92.63 (6)
O1^vii^—Na—P2	99.77 (6)	Na^iii^—O6—K^iv^	89.12 (6)
O6^viii^—Na—P2	151.35 (6)	P2—O7—Cu^vi^	124.98 (11)
O5—Na—P2	27.01 (4)	P2—O7—Na^vi^	99.93 (9)
O7^i^—Na—P2	85.78 (5)	Cu^vi^—O7—Na^vi^	104.36 (7)
O3^viii^—Na—P2	79.46 (5)	P2—O7—K^xii^	137.96 (10)
O5^i^—Na—P2	140.25 (5)	Cu^vi^—O7—K^xii^	90.92 (6)
O7^viii^—Na—P2	96.10 (4)	Na^vi^—O7—K^xii^	89.80 (6)
P2^i^—Na—P2	111.56 (3)	P2—O7—Na^iii^	84.73 (7)
P2^viii^—Na—P2	123.17 (4)	Cu^vi^—O7—Na^iii^	81.55 (6)
O1^vii^—Na—Cu^ix^	115.94 (6)	Na^vi^—O7—Na^iii^	167.87 (8)
O6^viii^—Na—Cu^ix^	86.18 (5)	K^xii^—O7—Na^iii^	79.43 (5)

Symmetry codes: (i) *x*+1, *y*, *z*; (ii) *x*+1/2, −*y*+3/2, *z*+1/2; (iii) *x*−1/2, −*y*+3/2, *z*+1/2; (iv) −*x*+3/2, *y*+1/2, −*z*+1/2; (v) −*x*+1, −*y*+1, −*z*+1; (vi) *x*−1, *y*, *z*; (vii) −*x*+1, −*y*+1, −*z*; (viii) *x*+1/2, −*y*+3/2, *z*−1/2; (ix) *x*−1/2, −*y*+3/2, *z*−1/2; (x) −*x*+3/2, *y*−1/2, −*z*+1/2; (xi) −*x*+1/2, *y*−1/2, −*z*+1/2; (xii) −*x*+1/2, *y*+1/2, −*z*+1/2.
